# Supplementary material for: First Evidence of Antibodies Against Lloviu Virus in Schreiber’s Bent-Winged Insectivorous Bats Demonstrate a Wide Circulation of the Virus in Spain
Source: Viruses. 2019 Apr 19;11(4):360. doi: 10.3390/v11040360 (PMC6521100; doi:10.3390/v11040360)
Supplement: Supplementary file 1 [file viruses-11-00360-s001.pdf]

Supplementary data - Production of LLOV Antigen for the Immunoblot assay

Recombinant transfer vectors

The C-terminal domain of the LLOV GP (GP_2, GenBank AN: JF828358) was recovered from bat LLOV samples and cloned into the pCRII vector by traditional TA cloning procedures. LLOV GP_2 DNA was re-amplified by PCR from the pCRII templates using the following forward and reverse primers, F-LL (5′ GCG CGA ATT CCG ATG CCA TTG GGA GGG TCT TCG 3′) and R-LL (5′ CGC GGT CGA CTC ATC GTG TTA TTC TGC ACA GAC AAA TCA AG 3′). Eco*R*1 and S*al*1 restriction sites are underlined, respectively. Primers were designed based on the nucleotide sequence of LLOV. Standard amplification conditions were applied: one cycle at 94 ºC for 2 min, followed by 40 cycles of 94 ºC for 1 min; 50ºC for 2 min 30 s; 72ºC for 1 min and a final extension of 72ºC for 10 min.

The 963 bp amplified GP_2 fragment was directionally subcloned into the Eco*R*I and S*al*I sites of pfastBac HT B donor plasmid (Invitrogen, cat nº10359-016) to construct a recombinant vector pfastbac-HIS-LLOV-GP_2 with a hexahistidine (6xHis) tag sequence. We used pfastbac-HT-CAT (chloramphenicol acetyltransferase gene, Invitrogen, cat nº10359-016) as control. pfastbac-HIS-LLOV-GP_2 was sequenced to rule out any mutation. Both pfastBac-HIS-LLOV-GP2 and pfastBac-HT-CAT were transformed into DH10bac *E. coli* respectively, for transposition into the bacmid (Invitrogen, cat nº10359-016). The recombinant bacmid DNA (Bacmid-6xHis-LLOV-GP_2), containing the entire LLOV GP_2 gene, and the control bacmid containing a CAT gene (Bacmid-6xHis-CAT), were isolated using anion exchange resin columns (Purelink HiPure Plasmid DNA Miniprep Kit, Invitrogen), screened by PCR and confirmed by sequencing.

Producing recombinant baculoviruses stock

Serial dilutions (10^{-4}, 10^{-5}, 10^{-6}, 10^{-7}) of the purified recombinant Bacmid-6xHis-LLOV-GP_2 and Bacmid-6xHis-CAT viral stocks were used to infect a *Spodoptera frugiperda* 21 (Sf21) insect cell line ($5x10^5$ cells/ml) to determine the viral titer. Single plaques were isolated to check for quality. Characterization was performed by PCR with the PUC/M13 Forward primer (5′ CCC AGT CAC GAC GTT GTA AAA CG 3′) and PUC/M13 Reverse primer (5′ AGC GGA TAA CAA TTT CAC ACA GG 3′) and standard conditions (Bac to Bac Expression System, Invitrogen Kit, cat n º 10359-016). Once clones were qualified, cells were infected as follows: $2x10^6$cells Sf21 cells were diluted in a supplemented Grace's Insect Medium, seeded in a 6-well plate, and incubated 1 hour at 27 ºC. Then, 100 µl of each viral stock was added to each well for 1 h adsorption at room temperature. Cells were incubated 4 days at 28 ºC or until demonstration of typical signs of late infection, when the cells and the supernatant was collected and transferred to sterile tubes. Tubes were centrifuged at 1000 xg for 5 min to remove cells and large debris. The clarified supernatants constituted the P1 viral stock. The titers were determined as $1.3x10^8$ pfu/ml for bacmid GP_2 and $1.4x10^9$ pfu/ml for bacmid CAT. Finally, the P1 stocks were amplified to obtain the working P2 viral stock by infecting 100 ml of Sf21 cells in culture ($0.5x10^6$ cells/ml) at a low multiplicity of infection (MOI, 0.5) with the corresponding baculovirus, as recommended (viral plate assay, Invitrogen, cat nº 10359-016). The final titers were $1.5x10^8$ pfu/ml and $1.75x10^8$ pfu/ml for the recombinant bacmids GP2 and CAT, respectively.

Production of LLOV GP_2 antigen

Sf21 insect cell suspensions ($1.0x10^8$ cells) were infected at a MOI of 5 with the recombinant Bacmid-6xHis-LLOV-GP_2 ($1.5x10^8$ pfu/ml) and with the control Bacmid-6xHis-CAT ($1.75x10^8$ pfu/ml), respectively. Both infections were incubated at 28°C for four days. Then, cultures were harvested and centrifuged at 12,000 rpm at 4°C for 10 min. Supernatant fractions were discarded and cell pellets lysed by addition of 1 ml of lysis buffer (cat. Nº 554778, BD) and 1µl of protease inhibitor cocktail per each $100x10^6$ cells (cat. 554779,

BD). Lysates were held on ice for 45 min and further sonicated (Bandelin Sonopuls, program: pulse on 20 seconds, 3 times). The supernatant (soluble fraction) and the pellet (insoluble fraction) were separated by centrifugation at 10.000 rpm for 10 min. The expression of recombinant 6xHis-LLOV-GP_2 and 6xHis-CAT proteins (40 and 28 KDa respectively) was analyzed in both (supernatant and pellet fractions) by Immunoblot using the monoclonal anti-His antibody (His Tag Mouse mAb HRP conjugate; cat. 9991s, Cell Signaling Technology, dilution 1:2500) as primary antibody, and the polyclonal anti-mouse IgG-HRP-link as a secondary antibody (cat. 7076; Cell Signaling Technology, dilution 1:2000). Protein visualization was performed using a peroxidase solution and DAB (3,3′-diaminobenzidine tetrahydrochloride) as substrate (CN/DAB substrate kit, thermo scientific, cat. 34000). The supernatant and pellet fractions were positive for both proteins (**Figure 1a**).

The recombinant 6xHis-LLOV-GP_2 protein (40 KDa) to be used as antigen in Immunoblot assays was obtained from a crude extract of the pellet fraction after its treatment to break up the inclusion bodies: the insoluble pellet of infected cells was resuspended in 500 µl of Inclusion Body Solubilization reagent (IBS, Thermo Fisher scientific). Recombinant 6xHis-CAT protein was obtained in the same conditions in order to have a negative control.
